# Prognostic analysis of radical resection for iCCA^phl^ and iCCA^pps^: A retrospective cohort study

**DOI:** 10.3389/fonc.2022.992606

**Published:** 2022-11-21

**Authors:** Zetao Yu, Qingqiang Ni, Hongtao Jia, Hengjun Gao, Faji Yang, Huaqiang Zhu, Fangfeng Liu, Jianlu Wang, Xu Zhou, Hong Chang, Jun Lu

**Affiliations:** ^1^ Department of Hepatobiliary Surgery, Shandong Provincial Hospital, Shandong University, Jinan, Shandong, China; ^2^ Department of Hepatobiliary Surgery, Shandong Provincial Hospital Affiliated to Shandong First Medical University, Jinan, Shandong, China

**Keywords:** iCCA, iCCA^pps^, iCCA^phl^, prognostic analysis, radical resection

## Abstract

**Backgroud:**

At present, there is no definitive conclusion about the relative prognostic factors on intrahepatic cholangiocarcinoma perihilar large duct type (iCCA^phl^) and iCCA peripheral small duct type (iCCA^pps^).

**Aim of the study:**

To compare the prognoses of two different types of iCCA, and identify the independent risk factors affecting the long-term survival of patients undergoing radical resection for iCCA.

**Methods:**

This study included 89 patients with iCCA who underwent radical resection at the Department of Hepatobiliary Surgery of the East Yard of the Shandong Provincial Hospital between January 2013 and March 2022. According to the tumor origin, these patients were divided into the iCCA^phl^ group (n = 37) and iCCA^pps^ group (n = 52). The prognoses of the two groups were compared using Kaplan–Meier analysis, whereas the independent risk factors of their prognoses were identified using Cox univariate and multivariate regression analyses.

**Results:**

In the iCCA^pps^ group, the independent risk factors for overall survival included diabetes history (p = 0.006), lymph node metastasis (p = 0.040), and preoperative carbohydrate antigen 19-9 (p = 0.035). In the iCCA^phl^ group, the independent risk factors for overall survival included multiple tumors (p = 0.010), tumor differentiation grade (p = 0.008), and preoperative jaundice (p = 0.009).

**Conclusions:**

Among the iCCA patients who underwent radical resection, the long-term prognosis of iCCA^pps^ maybe better than that of iCCA^phl^. The prognoses of these two types of iCCA were affected by different independent risk factors.

## Introduction

Malignancies of the biliary tract can be categorized as intrahepatic cholangiocarcinoma (iCCA), hilar cholangiocarcinoma, carcinoma of common bile duct, or gallbladder carcinoma according to the location of the tumor. Hilar cholangiocarcinoma originates from where the bilateral hepatic ducts join the cystic duct and common hepatic duct, whereas iCCA originates from the second-order bile ducts and above (1). The iCCA is the second most common hepatic malignancy after hepatocellular carcinoma and has shown an escalating incidence in recent years (2, 3). Although the pathophysiology, genetic and epigenetic aberrations have not been fully discovered, it is currently believed that its pathogenesis may be associated with primary sclerosing cholangitis, parasitic infection, cholelithiasis, alcoholic liver disease, non-specific cirrhosis, diabetes, or asbestos exposure (4–6). Due to its delayed presentation of symptoms, difficulty in diagnosis, relatively delayed treatment initiation, easy recurrence, and lack of effective treatment methods other than surgery, the prognosis of iCCA is poorer than that of hepatocellular carcinoma (7–9). Some studies have suggested that factors associated with the long-term prognosis of patients with iCCA include preoperative lymph node metastasis, carbohydrate antigen 19-9 positivity, vascular invasion, and number of tumors (10–12).

**Figure 1:**
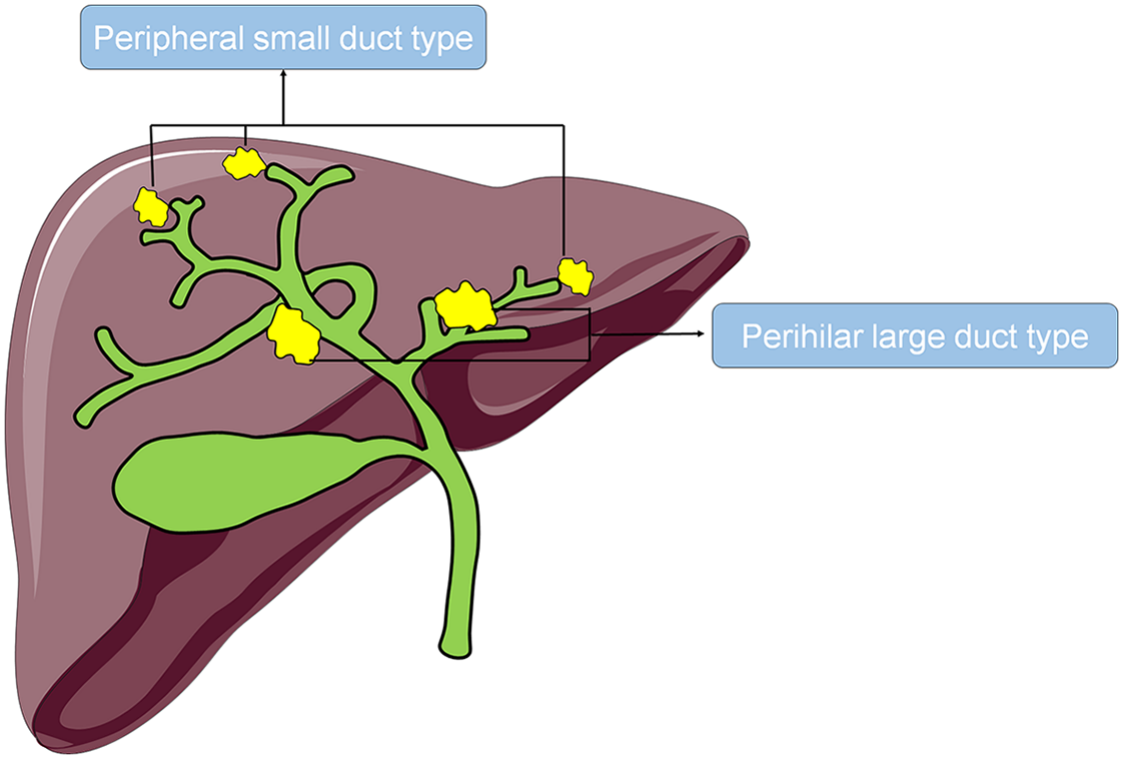
Tumor location of iCCA^phl^ and iCCA^pps^.

According to the origin of the tumor, iCCA can be categorized as the perihilar large duct type (iCCA^phl^) or the peripheral small duct type (iCCA^pps^). ICCA^phl^ refers to tumors originating from the large second-order bile ducts and above involving the peribiliary glands (**Figure 1**). They are composed of a large tubular or papillary proliferation of tall columnar epithelium with extracellular mucin production, often admixed with poorly differentiated carcinoma cells (13–16). On the other hand, iCCA^pps^ refers to tumors originating from the small bile ducts, such as Herling’s duct, which are usually smaller than the segmental branches (**Figure 1**). They are composed of a small tubular proliferation of small cuboidal epithelium with ductular pattern or closely packed cord-like structures, but lacking large glands with tall columnar cells (13–16). Previous studies have revealed several differences between these two subtypes in their histological characteristics, expression of interleukin 33, gene regulatory networks, and immune infiltration (17–20). However, the current research on the postoperative overall survival and disease-free survival or the associated risk factors of iCCA^pps^ and iCCA^phl^ is limited. Therefore, this study aimed to compare the long-term prognosis of the two subtypes of iCCA and investigate their corresponding risk factors to provide references for the clinical diagnosis and treatment of iCCA.

## Materials and methods

### Data collection

This study included patients with iCCA who underwent surgical resection at the Department of Hepatobiliary Surgery of the East Yard of the Shandong Provincial Hospital between January 2013 and March 2022. The inclusion criteria were as follows: (1) history of radical resection for iCCA; (2) iCCA diagnosis on postoperative pathological analysis; and (3) availability of computed tomography (CT) or magnetic resonance imaging (MRI) data of the liver. The exclusion criteria were as follows: (1) only surgical or needle biopsy performed; (2) incomplete CT or MRI data; and (3) no follow-up information collected after discharge. In total, 89 patients were included, who were subsequently divided into the iCCA^phl^ group (n = 37) and the iCCA^pps^ group (n = 52).

The demographic information of the patients was first collected during hospitalization. This included age, sex, length of hospitalization, drinking history, history of underlying diseases (hypertension, hyperglycemia, coronary heart disease, or history of abdominal surgery), preoperative jaundice, vascular invasion, number of tumors, surgical margin, maximum tumor diameter (>5 cm), tumor differentiation grade, lymph node metastasis, iCCA classification, preoperative carcinoembryonic antigen level, preoperative carbohydrate antigen 19-9 level, preoperative alpha-fetoprotein level, liver cirrhosis, intrahepatic bile duct stones, hepatitis B infection, use of blood transfusion products during surgery, surgical classification (whether >2 liver segments were removed), anesthesia classification, perioperative blood transfusion products, and postoperative chemotherapy. Furthermore, the overall survival and disease-free survival were recorded by following up on the telephone. The follow-up period ended either on May 1, 2022 or when the patient died or was lost to follow-up. This study was approved by the Ethics Committee of the Shandong Provincial Hospital, and informed consent was waived for all participants.

### Outcome definition

Overall survival was defined as the time from surgery to death or when follow-up ended. Disease-free survival was defined as the time from surgery to the first discovery of recurrence or when follow-up ended. Patients were allocated to the iCCA^phl^ group if their imaging findings revealed that the tumor was located in the large intrahepatic second-order bile ducts and above or was accompanied by the dilation of the distal bile ducts, and/or their histopathology revealed that the tumor was composed of a tubular or papillary component with tall columnar epithelium. Patients with tumors originating from branches other than the aforementioned branches were allocated to the iCCA^pps^ group, and/or their histopathology revealed that the tumor was composed of small tubules with cuboidal epithelium.

### Statistical analysis

Statistical Product and Service Solutions, version 26 (IBM Corp, Armonk, New York), software was adopted for all statistical analyses. Pairwise comparison was performed between the two groups of iCCA patients. Variables conforming to normal distribution, variables not conforming to normal distribution, and categorical variables were analyzed using the student’s t-test, Mann–Whitney U test, and Fisher’s exact test, respectively; P-values <0.05 were considered statistically significant. The median overall survival, 1-year and 3-year survival rates, median disease-free survival, and 1-year and 3-year recurrence-free rates were calculated using the Kaplan–Meier analysis, followed by plotting a survival curve. Moreover, Cox univariate regression analysis was performed for the iCCA^phl^ and iCCA^pps^ groups. Indicators with statistically significant differences identified in the univariate analysis (P < 0.05) were subsequently included in the Cox multivariate regression model to investigate the independent risk factors affecting the postoperative overall survival and disease-free survival of the patients of the two groups.

## Results

### Pairwise comparison of the two groups

The iCCA^phl^ group included 37 patients (average age, 60.35 ± 9.85 years), comprising 18 (48.6%) male patients. The iCCA^pps^ group included 52 patients (average age, 59.33 ± 9.8 years), comprising 27 (51.9%) male patients. The analysis of the demographic data revealed no significant differences in the age (60.35 ± 9.85 vs. 59.33 ± 9.80), man (18 [48.6%] vs. 27 [51.9%]), drinking history (10 [27.0%] vs. 18 [34.6%]), and history of diabetes (4 [10.8%] vs. 9 [17.3%]), hypertension (4 [10.8%] vs. 9 [17.3%]), coronary heart disease (4 [10.8%] vs. 3 [5.8%]), or abdominal surgery (8 [21.6%] vs. 6 [11.5%]) between the two groups.

**Figure 2:**
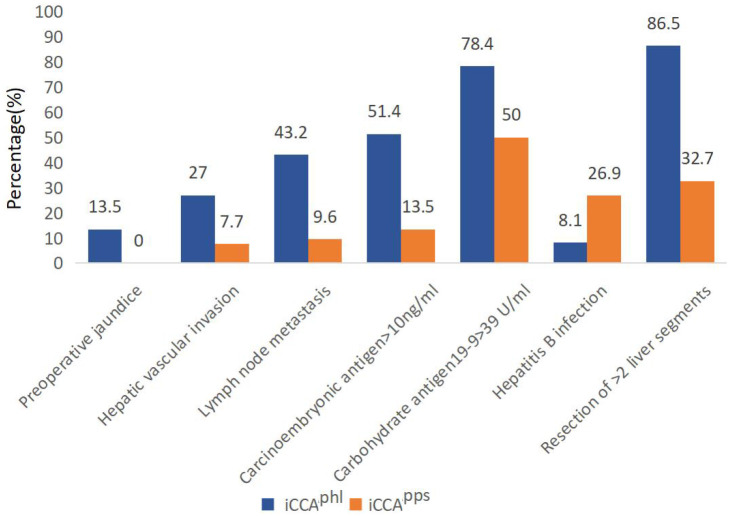
Pairwise comparison of the two types of intrahepatic cholangiocarcinoma (P < 0.05). The bars in the graph indicate the number of cases in each group for each risk factor. iCCA^phl^, perihilar large duct type of intrahepatic cholangiocarcinoma; iCCA^pps^, peripheral small duct type of intrahepatic cholangiocarcinoma.

The average length of hospitalization differed significantly between the iCCA^phl^ and iCCA^pps^ groups (13.00 [9.50–19.00] vs. 11.00 [9.00–13.75]). Moreover, the iCCA^phl^ and iCCA^pps^ groups showed significant differences in the proportion of cases with preoperative jaundice (5 [13.5%] vs. 0 [0.0%]), hepatic vascular invasion (10 [27.0%] vs. 4 [7.7%]), lymph node metastasis (16 [43.2%] vs. 5 [9.6%]), preoperative carcinoembryonic antigen >10 ng/mL (19 [51.4%] vs. 7 [13.5%]), preoperative carbohydrate antigen 19-9 >39 U/mL (29 [78.4%] vs. 26 [50.0%]), hepatitis B infection (8 [8.1%] vs. 14 [26.9%]), and resection of >2 liver segments (32 [86.5%] vs. 17 [32.7%]). However, the remaining indicators did not show significant pairwise differences (**Table 1**; **Figure 2**).

**Table 1:** Pairwise comparison of the two types of intrahepatic cholangiocarcinoma.

Variable	iCCA^phl^ (n = 37)	iCCA^pps^ (n = 52)	P-value
Length of hospitalization (days)	13.00 (9.50,19.00)	11.00 (9.00,13.75)	0.029
Preoperative jaundice	5 (13.5%)	0 (0.0%)	0.011
Vascular invasion	10 (27.0%)	4 (7.7%)	0.018
Lymph node metastasis	16 (43.2%)	5 (9.6%)	<0.001
Carcinoembryonic antigen >10 ng/mL	19 (51.4%)	7 (13.5%)	<0.001
Carbohydrate antigen 19-9 >39 U/mL	29 (78.4%)	26 (50.0%)	0.008
Hepatitis B infection	8 (8.1%)	14 (26.9%)	0.030
Resection of >2 liver segments	32 (86.5%)	17 (32.7%)	<0.001

(iCCA^phl^, perihilar large duct type of intrahepatic cholangiocarcinoma; iCCA^pps^, peripheral small duct type of intrahepatic cholangiocarcinoma).

**Figure 3:**
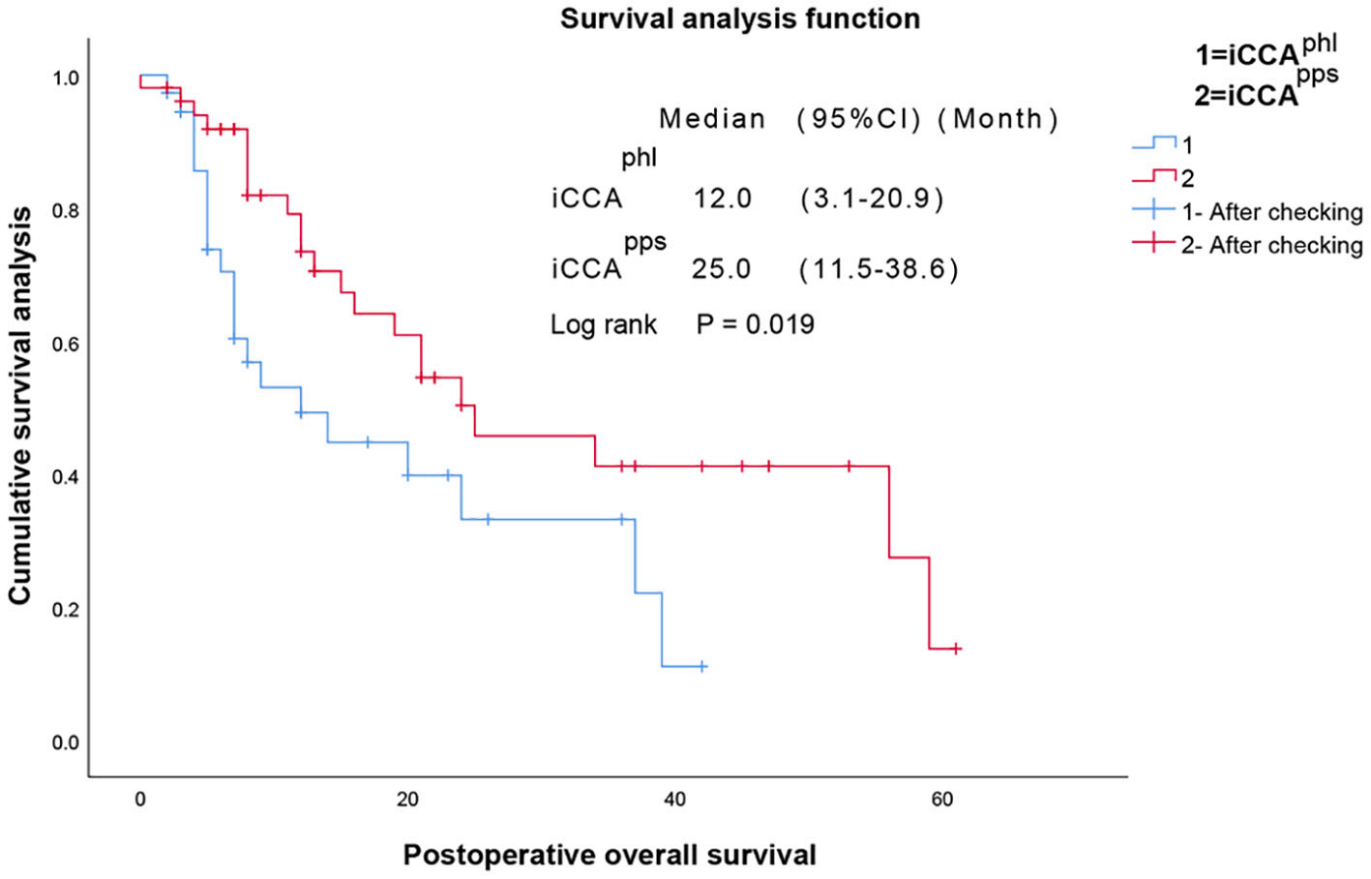
Pairwise comparison of the overall survival between the two types of intrahepatic cholangiocarcinoma. iCCA^phl^, perihilar large duct type of intrahepatic cholangiocarcinoma; iCCA^pps^, peripheral small duct type of intrahepatic cholangiocarcinoma; M [95% CI], median [95% confidence interval].

**Figure 4:**
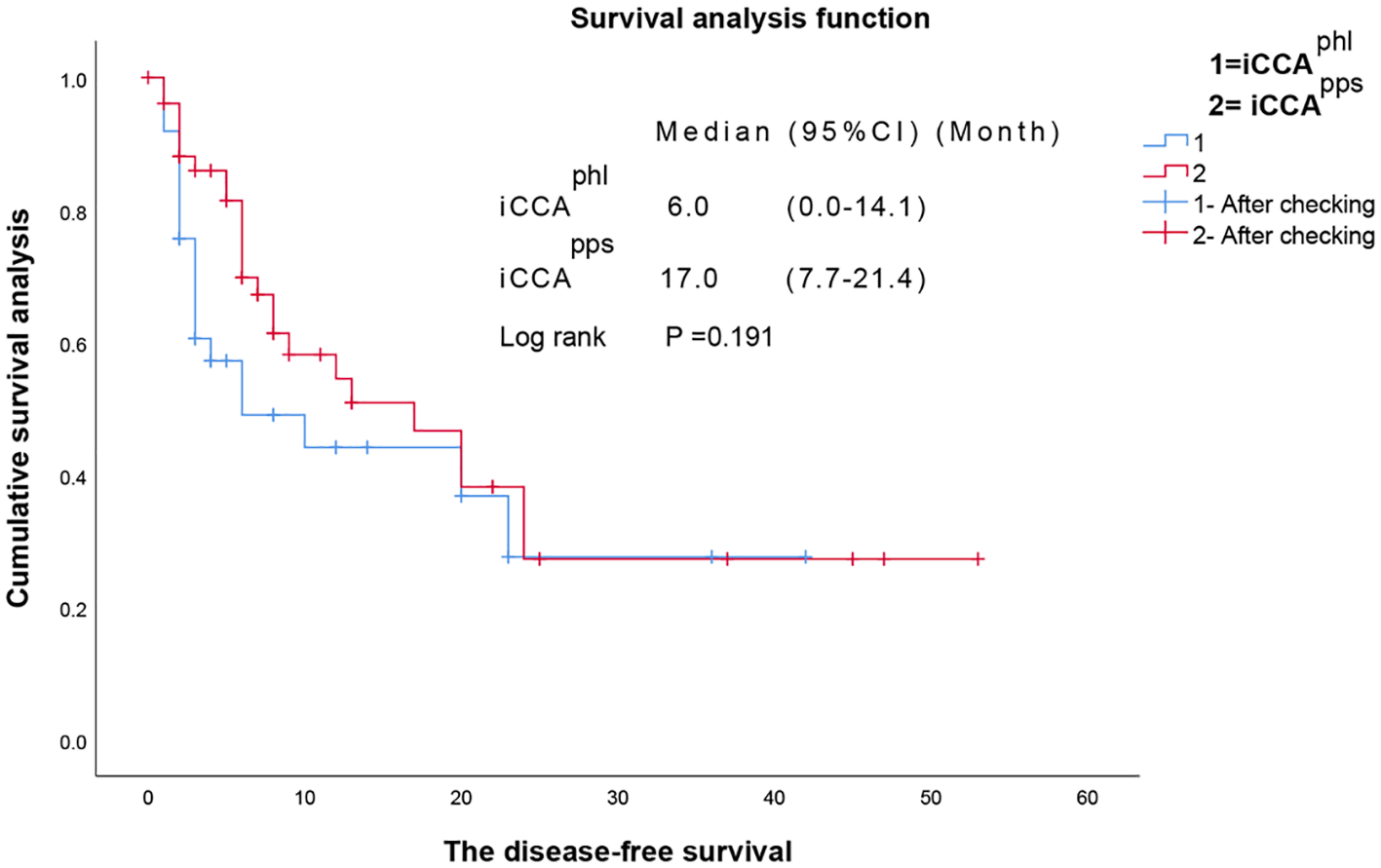
Pairwise comparison of the disease-free survival between the two types of intrahepatic cholangiocarcinoma. iCCA^phl^, perihilar large duct type of intrahepatic cholangiocarcinoma; iCCA^pps^, peripheral small duct type of intrahepatic cholangiocarcinoma; CI, confidence interval.

Kaplan–Meier analysis revealed that the median overall survival in the iCCA^phl^ group was 12.0 (95% confidence interval [CI]: 3.09–20.91) months, and the 1-year and 3-year overall survival rates were 49.3% and 33.2%, respectively. In contrast, the median overall survival in the iCCA^pps^ group was 25.0 (95% CI: 11.45–38.55) months, with 1-year and 3-year overall survival rates of 73.5%, and 41.2%, respectively. Additionally, the pairwise differences between the two groups were statistically significant (P = 0.019) (**Figure 3**). It showed that the hazard ratio (HR) of the two tumor subtypes was 1.780, 95% CI: 0.770–4.113 and P=0.177 after adjustment with multivariate cox regression analysis of the variables with statistical differences in univariate analysis and the two tumor subtypes. The median disease-free survival in the iCCA^phl^ group was 6.0 (95% CI: 0.00–14.12) months, and the 1-year and 3-year recurrence-free rates were 44.1% and 27.6%, respectively. The median disease-free survival in the iCCA^pps^ group was 17.0 (95% CI: 7.65–26.35) months, with 1-year and 3-year recurrence-free rates of 54.5% and 27.2%, respectively. However, pairwise differences between the two groups were not significant (P = 0.191) (**Figure 4**).

### Risk factor analysis of the prognosis of radical resection for iCCA^pps^

Cox univariate regression analysis of the overall survival in the iCCA^pps^ group revealed that length of hospitalization, age, diabetes history, vascular invasion, multiple tumors, lymph node metastasis, preoperative carbohydrate antigen 19-9 >39 U/mL, intrahepatic bile duct stones, surgical resection of >2 liver segments, postoperative complication grade, and hepatitis B infection were the risk factors. Subsequent multivariate regression analysis showed that diabetes history (HR: 11.768, 95% CI: 2.003–69.136), lymph node metastasis (HR: 6.180, 95% CI: 1.090–35.038), and preoperative carbohydrate antigen 19-9 >39 U/mL (HR: 3.140, 95% CI: 1.081–9.121) were independent risk factors. Additionally, length of hospitalization (HR: 1.287, 95% CI: 1.060–1.562) exhibited a statistically significant effect (**Table 2**).

**Table 2:** Risk factor analysis of the prognosis of radical resection for the two types of intrahepatic cholangiocarcinoma.

Variables	Univariate analysis		Multivariate analysis	
	HR (95%CI)	Pvalue	HR (95%CI)	Pvalue
**Peripheral small duct type of intrahepatic cholangiocarcinoma**				
Length of hospitalization	1.196 (1.025-1.394)	0.023	1.287 (1.060-1.562)	0.011
Age	1.054 (1.005-1.106)	0.030		
Diabetes history	3.171 (1.017-9.891)	0.047	11.768 (2.003-69.136)	0.006
Vascular invasion	4.095 (1.121-14.955)	0.033		
Multiple tumors	4.078 (1.540-10.801)	0.005		
Lymph node metastasis	6.617 (1.997-21.931)	0.002	6.180 (1.090-35.038)	0.040
Elevated carbohydrate antigen 19-9 levels	2.502 (1.042-6.005)	0.040	3.140 (1.081-9.121)	0.035
Intrahepatic bile duct stones	3.767 (1.049-13.534)	0.042		
Hepatitis B infection	0.171 (0.039-0.750)	0.019		
Resection of >2 liver segments	4.409 (1.737-11.193)	0.002		
Complication grade	1.892 (1.088-3.291)	0.024		
**Perihilar large duct type of intrahepatic cholangiocarcinoma**				
Multiple tumors	5.821 (1.555-21.795)	0.009	6.246 (1.552-25.140)	0.010
Tumor differentiation grade	2.988 (1.262-7.072)	0.013	3.522 (1.384-8.963)	0.008
Lymph node metastasis	2.642 (1.078-6.474)	0.034		
Preoperative jaundice	4.907 (1.559-15.441)	0.007	4.883 (1.496-15.944)	0.009

Cox univariate regression analysis of the disease-free survival in the iCCA^pps^ group showed that lymph node metastasis (HR: 11.496, 95% CI: 2.962–44.623) and preoperative carbohydrate antigen 19-9 >39 U/mL (HR: 3.031, 95% CI: 1.322–6.946) were risk factors. Subsequent Cox multivariate analysis suggested that both these were independent risk factors for the disease-free survival in the iCCA^pps^ group (lymph node metastasis, HR: 7.765, 95% CI: 1.961–30.750; preoperative carbohydrate antigen 19-9 >39 U/mL, HR: 2.555, 95% CI: 1.074–6.082).

### Risk factor analysis of the prognosis of radical resection for iCCA^phl^

Cox univariate regression analysis showed that the risk factors affecting the overall survival in the iCCA^phl^ group were multiple tumors, tumor differentiation grade, lymph node metastasis, and preoperative jaundice. Subsequent multivariate analysis showed that multiple tumors (HR: 6.246, 95% CI: 1.552–25.140), tumor differentiation grade (HR: 3.522, 95% CI: 1.384–8.963), and preoperative jaundice (HR: 4.883, 95% CI: 1.496–15.944) were independent risk factors for the overall survival of patients with iCCA^phl^ (**Table 2**).

Furthermore, Cox univariate regression analysis showed that preoperative jaundice (HR: 4.498, 95% CI: 1.540–13.134) was the only risk factor of disease-free survival in the iCCA^phl^ group.

## Discussion

Recently, the incidence of iCCA has been on the rise. Moreover, the diagnosis may be delayed due to its hidden onset in most cases, and the window for surgery may have already been missed due to the severe disease progression, thereby leading to poor prognosis. Multiple studies have reported that elevated preoperative carbohydrate antigen 19-9 and carcinoembryonic antigen levels and lymph node metastasis are important prognostic indicators in iCCA patients (21–25). Recent studies have shown that iCCA can be categorized as iCCA^phl^ and iCCA^pps^, and these two subtypes differ significantly in their pathological morphology and survival prognosis (14, 15, 26). Our study showed that the length of hospitalization was longer and the incidence of preoperative jaundice, vascular invasion, lymph node metastasis, and elevated preoperative carcinoembryonic antigen and carbohydrate antigen 19-9 levels were more common in the iCCA^phl^ patients than in the iCCA^pps^ patients. In univariable analysis, the median postoperative overall survival was shorter in the iCCA^phl^ group than in the iCCA^pps^ group. However, after adjustment with multivariate cox regression analysis, it revealed no significant differences in postoperative survival time between the two tumor subtypes. This may be related to the low incidence of the disease and the low number of patients enrolled. Therefore, further large-sample prospective studies are needed to identify the differences in prognosis between iCCA^phl^ and iCCA^pps^. Therefore, it was speculated that iCCA^phl^ maybe more aggressive than iCCA^pps^ and results in a poor overall survival, which corroborated the findings of Shinichi et al. (26). Thus, in clinical practice, it would be possible to predict the patient’s prognosis early based on the preoperative imaging findings. Subsequently, early interventions can be introduced for the treatment of iCCA^phl^, thereby reducing the risk of postoperative recurrence and prolonging the overall survival of the patient.

Currently, differentiation of the iCCA types has not been widely implemented clinically. The Cox multivariate regression analysis in this study suggested that the length of hospitalization, diabetes history, lymph node metastasis, and elevated carbohydrate antigen 19-9 levels were independent risk factors for the prognosis of iCCA^pps^, whereas multiple tumors, tumor differentiation, and preoperative jaundice were independent risk factors for the prognosis of iCCA^phl^. Since these two subtypes have completely different risk factors, prognosis prediction based solely on the risk factors for iCCA may not match the actual situation and could be inaccurate. Therefore, it is necessary to clinically differentiate between iCCA^pps^ and iCCA^phl^, evaluate the corresponding risk factors, predict the prognosis early, and introduce appropriate and timely treatment.

This study found that the effect of hepatitis B infection on the patients’ overall survival was statistically significant only in the univariate analysis, but not in the multivariate analysis. Currently, the effect of hepatitis B infection on the prognosis of iCCA remains controversial. A study by Jeong et al. indicated that hepatitis B infection was a strong predictor of the good prognosis of iCCA (27, 28), whereas Ahn et al. found that it was not an independent prognostic factor of iCCA (29, 30). Therefore, the influence of hepatitis B infection on the prognosis of iCCA requires further verification *via* prospective studies.

The strength of this study is that both univariate and multivariate cox regression analyses were performed for the two types of iCCA to identify the differences in their prognostic factors. Moreover, unlike the study by Shinichi et al., tumors >5 cm in size were included in this study, thereby increasing the differentiation by tumor size and consequently reducing the selection bias caused by deliberately excluding large-sized tumors. The limitation of this study is that this was a single-center study, and only patients admitted to the Department of Hepatobiliary Surgery of the East Yard of the Shandong Provincial Hospital were included. Furthermore, only those patients who underwent radical resection were evaluated; therefore, further investigations are warranted to conclude whether the findings of this study are applicable to iCCA patients who do not undergo surgery.

In conclusion, this study showed that iCCA^phl^ maybe more aggressive than iCCA^pps^, and the overall survival maybe differ between them. In clinical practice, individualized early interventions should be introduced in patients with iCCA according to the disease subtype to improve their prognosis.

## Data availability statement

The raw data supporting the conclusions of this article will be made available by the authors, without undue reservation.

## Ethics statement

The studies involving human participants were reviewed and approved by Ethics Committee of Shandong Provincial Hospital. Written informed consent for participation was not required for this study in accordance with the national legislation and the institutional requirements.

## Author contributions

ZY: Writing-original draf, data curation. QN: Investigation, methodology, writing review and editing, conceptualization. HJ: Writing review and editing. HG: Investigation, methodology, writing review and editing. FY: Methodology, writing review and editing. HZ: Investigation, methodology, writing review and editing. FL: Investigation, methodology, conceptualization. JW: Investigation, supervision. XZ: Investigation, supervision, methodology. JL: Investigation, conceptualization, methodology, supervision. HC: Investigation, conceptualization, methodology, supervision. All authors had full access to all the data in the study and had final responsibility for the decision to submit for publication. All authors contributed to the article and approved the submitted version.

## Funding

This study was supported by Shandong Provincial Natural Science Foundation, Grant/Award Number: ZR2020MH054.

## Conflict of interest

The authors declare that the research was conducted in the absence of any commercial or financial relationships that could be construed as a potential conflict of interest.

## Publisher’s note

All claims expressed in this article are solely those of the authors and do not necessarily represent those of their affiliated organizations, or those of the publisher, the editors and the reviewers. Any product that may be evaluated in this article, or claim that may be made by its manufacturer, is not guaranteed or endorsed by the publisher.

## References

[1] El-Diwany R Pawlik TM Ejaz A. Intrahepatic cholangiocarcinoma. Surg Oncol Clin N Am (2019) 28:587–99. doi: 10.1016/j.soc.2019.06.002 31472907

[2] Entezari P Riaz A. Intrahepatic cholangiocarcinoma. Semin Intervent Radiol (2020) 37:475–83. doi: 10.1055/s-0040-1719188 PMC7732564 33328703

[3] Endo I Gonen M Yopp AC Dalal KM Zhou Q Klimstra D. Intrahepatic cholangiocarcinoma: Rising frequency, improved survival, and determinants of outcome after resection. Ann Surg (2008) 248:84–96. doi: 10.1097/SLA.0b013e318176c4d3 18580211

[4] Brandi G Tavolari S. Asbestos and intrahepatic cholangiocarcinoma. Cells (2020) 9(2):421. doi: 10.3390/cells9020421 32059499 PMC7072580

[5] Chapman MH Webster GJ Bannoo S Johnson GJ Wittmann J Pereira SP. Cholangiocarcinoma and dominant strictures in patients with primary sclerosing cholangitis: A 25-year single-centre experience. Eur J Gastroenterol Hepatol (2012) 24:1051–8. doi: 10.1097/MEG.0b013e3283554bbf PMC3584158 22653260

[6] Shaib YH El-Serag HB Davila JA Morgan R McGlynn KA. Risk factors of intrahepatic cholangiocarcinoma in the United States: A case-control study. Gastroenterology (2005) 128:620–6. doi: 10.1053/j.gastro.2004.12.048 15765398

[7] Nathan H Aloia TA Vauthey JN Abdalla EK Zhu AX Schulick RD. A proposed staging system for intrahepatic cholangiocarcinoma. Ann Surg Oncol (2009) 16:14–22. doi: 10.1245/s10434-008-0180-z 18987916

[8] Kelley RK Bridgewater J Gores GJ Zhu AX. Systemic therapies for intrahepatic cholangiocarcinoma. J Hepatol (2020) 72:353–63. doi: 10.1016/j.jhep.2019.10.009 31954497

[9] Bekki Y Von Ahrens D Takahashi H Schwartz M Gunasekaran G. Recurrent intrahepatic cholangiocarcinoma - review. Front Oncol (2021) 11:776863. doi: 10.3389/fonc.2021.776863 34746017 PMC8567135

[10] Lurje G Bednarsch J Czigany Z Lurje I Schlebusch IK Boecker J. The prognostic role of lymphovascular invasion and lymph node metastasis in perihilar and intrahepatic cholangiocarcinoma. Eur J Surg Oncol (2019) 45:1468–78. doi: 10.1016/j.ejso.2019.04.019 31053477

[11] Asaoka T Kobayashi S Hanaki T Iwagami Y Tomimaru Y Akita H. Clinical significance of preoperative CA19-9 and lymph node metastasis in intrahepatic cholangiocarcinoma. Surg Today (2020) 50:1176–86. doi: 10.1007/s00595-020-01992-x 32221659

[12] Wang Y Li J Xia Y Gong R Wang K Yan Z. Prognostic nomogram for intrahepatic cholangiocarcinoma after partial hepatectomy. J Clin Oncol (2013) 31:1188–95. doi: 10.1200/JCO.2012.41.5984 23358969

[13] Yamada M Yamamoto Y Sugiura T Kakuda Y Ashida R Tamura S. Comparison of the clinicopathological features in small bile duct and bile ductular type intrahepatic cholangiocarcinoma. Anticancer Res (2019) 39:2121–7. doi: 10.21873/anticanres.13325 30952758

[14] Aishima S Oda Y. Pathogenesis and classification of intrahepatic cholangiocarcinoma: Different characters of perihilar large duct type versus peripheral small duct type. J Hepatobil Pancreat Sci (2015) 22:94–100. doi: 10.1002/jhbp.154 25181580

[15] Nakanuma Y Sato Y Harada K Sasaki M Xu J Ikeda H. Pathological classification of intrahepatic cholangiocarcinoma based on a new concept. World J Hepatol (2010) 2:419–27. doi: 10.4254/wjh.v2.i12.419 PMC3010511 21191517

[16] Kendall T Verheij J Gaudio E Evert M Guido M Goeppert B. Anatomical, histomorphological and molecular classification of cholangiocarcinoma. Liver Int (2019) 39 Suppl 1:7–18. doi: 10.1111/liv.14093 30882996

[17] Song G Shi Y Meng L Ma J Huang S Zhang J. Single-cell transcriptomic analysis suggests two molecularly subtypes of intrahepatic cholangiocarcinoma. Nat Commun (2022) 13:1642. doi: 10.1038/s41467-022-29164-0 35347134 PMC8960779

[18] Sawada R Ku Y Akita M Otani K Fujikura K Itoh T. Interleukin-33 overexpression reflects less aggressive tumour features in large-duct type cholangiocarcinomas. Histopathology (2018) 73:259–72. doi: 10.1111/his.13633 29675965

[19] Akita M Sofue K Fujikura K Otani K Itoh T Ajiki T. Histological and molecular characterization of intrahepatic bile duct cancers suggests an expanded definition of perihilar cholangiocarcinoma. HPB (Oxford) (2019) 21:226–34. doi: 10.1016/j.hpb.2018.07.021 30170977

[20] Yamashita YI Wang H Kurihara T Tsujita E Nishie A Imai K. Clinical significances of preoperative classification of intrahepatic cholangiocarcinoma: Different characteristics of perihilar vs. peripheral ICC. Anticancer Res (2016) 36:6563–9. doi: 10.21873/anticanres.11260 27919984

[21] Juntermanns B Kaiser GM Itani Gutierrez S Heuer M Buechter M Kahraman A. CA19-9 in intrahepatic cholangiocarcinoma: A diagnostic and prognostic armamentarium? Chirurg (2018) 89:466–71. doi: 10.1007/s00104-018-0636-z 29644426

[22] Moro A Mehta R Sahara K Tsilimigras DI Paredes AZ Farooq A. The impact of preoperative CA19-9 and CEA on outcomes of patients with intrahepatic cholangiocarcinoma. Ann Surg Oncol (2020) 27:2888–901. doi: 10.1245/s10434-020-08350-8 32198569

[23] Jolissaint JS Soares KC Seier KP Kundra R Gönen M Shin PJ. Intrahepatic cholangiocarcinoma with lymph node metastasis: Treatment-related outcomes and the role of tumor genomics in patient selection. Clin Cancer Res (2021) 27:4101–8. doi: 10.1158/1078-0432.CCR-21-0412 PMC8282702 33963001

[24] Ji GW Zhu FP Zhang YD Liu XS Wu FY Wang K. A radiomics approach to predict lymph node metastasis and clinical outcome of intrahepatic cholangiocarcinoma. Eur Radiol (2019) 29:3725–35. doi: 10.1007/s00330-019-06142-7 30915561

[25] Li H Feng Y Liu C Li J Li J Wu H. Importance of normalization of carbohydrate antigen 19-9 in patients with intrahepatic cholangiocarcinoma. Front Oncol (2021) 11:780455. doi: 10.3389/fonc.2021.780455 35004301 PMC8728073

[26] Shinichi A Yousuke K Yunosuke N Tomohiro I Kenichi T Akinobu T. Proposal of progression model for intrahepatic cholangiocarcinoma: Clinicopathologic differences between hilar type and peripheral type. Am J Surg Pathol (2007) 31(7):1059–67. doi: 10.1097/PAS.0b013e31802b34b6 17592273

[27] Zhou HB Hu JY Hu HP. Hepatitis b virus infection and intrahepatic cholangiocarcinoma. World J Gastroenterol (2014) 20:5721–9. doi: 10.3748/wjg.v20.i19.5721 PMC4024782 24914333

[28] Jeong S Luo G Wang ZH Sha M Chen L Xia Q. Impact of viral hepatitis b status on outcomes of intrahepatic cholangiocarcinoma: A meta-analysis. Hepatol Int (2018) 12:330–8. doi: 10.1007/s12072-018-9881-y 29947010

[29] Seo JW Kwan BS Cheon YK Lee TY Shim CS Kwon SY. Prognostic impact of hepatitis b or c on intrahepatic cholangiocarcinoma. Korean J Intern Med (2020) 35:566–73. doi: 10.3904/kjim.2018.062 PMC7214366 31916422

[30] Ahn CS Hwang S Lee YJ Kim KH Moon DB Ha TY. Prognostic impact of hepatitis b virus infection in patients with intrahepatic cholangiocarcinoma. ANZ J Surg (2018) 88:212–7. doi: 10.1111/ans.13753 27598539

